# Clinical application of liquid biopsy in endometrial carcinoma

**DOI:** 10.1007/s12032-023-01956-4

**Published:** 2023-02-09

**Authors:** Yan Shen, Rui Shi, Rong Zhao, Hongbo Wang

**Affiliations:** 1 Health Management Center, Union Hospital, Tongji Medical College, Huazhong University of Science and Technology, 1277 Jiefang Avenue, Jianghan, Wuhan, 430022 Hubei China; 2 Tongji Medical College, Huazhong University of Science and Technology, 1095 Jiefang Avenue, Qiaokou, Wuhan, 430030 Hubei China; 3 Department of Obstetrics and Gynecology, Union Hospital, Tongji Medical College, Huazhong University of Science and Technology, 1277 Jiefang Avenue, Jianghan, Wuhan, 430022 Hubei China

**Keywords:** Endometrial cancer, Liquid biopsy, Circulating tumor cells, Circulating tumor DNA

## Abstract

Endometrial cancer is the most common gynecological malignant tumor in women, and its morbidity and mortality have been rising in recent years. Over the past two decades, the diagnosis, prognosis, and therapeutic strategies for endometrial cancer have not significantly improved, and reliable biomarkers for detecting and monitoring EC recurrence and progression remain limited. Tumor genome analysis identified molecular alterations related to the growth and progression of endometrial cancer, but these data are incomplete. Recently, through extensive exploration of liquid biopsy, it has been determined that circulating tumor cells and circulating tumor DNA can lay a foundation for real-time and non-invasive monitoring of tumors and provide novel insights into cancer evolution, invasion, and metastasis. Hence, this review aimed to analyze the value of liquid biopsy in endometrial cancer screening, early diagnosis, treatment response, and prognosis monitoring in order to prolong the survival time of EC patients.

Endometrial cancer (EC) is the most common gynecologic malignant tumor in women, with the fourth highest incidence rate after breast cancer, lung cancer, and colorectal cancer [1]. Moreover, it is the seventh most common malignant disease in the world [2]. In view of the rising obesity rate, the incidence of EC is anticipated to increase to the epidemic rate in the next few decades. So far, since the early 1990s, its incidence has increased by approximately 50% and is expected to rise to 33/100000 by 2035 [3].

At present, there is no screening method for EC for the general population. Endometrial sampling can lead to discomfort, bleeding, infection, and uterine perforation, and a biopsy may not be sufficient to make a diagnosis in up to 25% of cases [4]. Circulating tumor DNA (ctDNA) in liquid biopsy has been established as a valuable biomarker for the early diagnosis and monitoring of cancer and has been extensively studied in breast cancer, colorectal cancer, prostate cancer, lung cancer, and other malignancies [5–8]. It can identify both gene and epigenetic mutations and can be detected in plasma and urine. Herein, we reviewed the potential clinical application of liquid biopsy for the diagnosis and treatment of EC.

## CtDNA (Circulating-tumor DNA)

### Development history

CtDNA is a component of tumor-specific cell-free DNA (cfDNA) derived from malignant tumor cells. It comprises extracellular nucleic acid fragments and carries tumor-related genes. It typically results from tumor cell necrosis or apoptosis and can also be actively released into the plasma by DNA [9, 10]. In cancer patients, the level of ctDNA tremendously varies and may be as low as 0.01% or as high as 10% [11].

In 1948, Mandel and Metais first described the free circulating nucleic acid in human plasma, namely cfDNA [12], which represents all DNA in blood circulation. By 1977, Leon’s team discovered that the level of free DNA in cancer patients was higher than that in healthy controls [13]. Mutated oncogenes are common drivers of tumor growth. This discovery is the key to conceptually understanding that high-level circulating DNA in cancer patients is actually the source of tumors. From the discovery of the first oncogene SRC to the later oncogenes EGFR, NRAS/KRAS, and the presence of mutant KRAS fragments in the plasma of pancreatic cancer patients, these oncogenes have proved the real ctDNA for the first time. All major types of gene alterations in tumor tissues have been detected in ctDNA, including point mutations, copy number variations, chromosomal rearrangements, epigenetic changes, insertions, and deletions.

### Detection

At present, tissue biopsy remains the gold standard for the diagnosis of malignant tumors; nevertheless, its development is limited by the risks of puncture injury, tumor metastasis, bleeding, and infection. Compared with traditional tissue biopsy, genome analysis using blood, plasma, or other body fluid samples is less invasive to patients; besides, it can reveal detailed genetic alterations in the tumor. Furthermore, it is convenient to repeat sampling so as to dynamically monitor the molecular fluctuations in tumors in real time. Monitoring cfDNA levels has the potential to detect tumors that are not discernible or uncertain via imaging examination, such as residual tumors following resection.

In the early 1990s, allele-specific PCR technology was first introduced for the measurement of cfDNA [14]. With advances in detection technology, they are currently divided into two categories: PCR-based technology and Nest generation sequencing (NGS)-based technology.

QPCR typically utilizes quenched fluorescent probes and has high specificity. It only needs to calculate the number of reactions containing wild-type or mutant PCR products to obtain sensitive and accurate quantification, but its repeatability is low. Therefore, a new technology based on qPCR has been developed.

Beaming (beads, extrusion, amplification, and magnetism) is a digital PCR (dPCR) approach that combines PCR and flow cytometry. The amplified target DNA is isolated by magnetic purification and opening of the emulsion, identified with a hybrid fluorescent probe, and counted by flow cytometry. Regrettably, only a few laboratories and commercial entities are able to effectively use this technology.

The extensively used ddPCR technology combines qPCR and beaming. DdPCR involves quenching fluorescent DNA probes, PCR components, and lotions of sample DNA. Compared with beaming and qPCR, ddPCR is a relatively inexpensive method and provides enhanced performance indicators.

NGS is technically complex but is highly scalable and can detect rare or unknown mutations. Nonetheless, despite this method being able to detect mutant alleles with a frequency as low as 0.1%, its error rate cannot be overlooked. Therefore, the standard version of this technique may not be sufficient to analyze low amounts of ctDNA, such as ctDNA of patients with early-stage cancer or postoperative patients.

### Clinical application of ctDNA

In 2010, Dobrzycka et al. demonstrated the feasibility of cfDNA in EC by detecting p53 and KRAS mutations in the plasma of patients with early serous EC and type 2 endometrioid adenocarcinoma for the first time [15]. Later, another study determined that the level of cfDNA in G2/G3 EC patients was higher than that in G1 EC patients [16].

### Early diagnosis

Studies on ctDNA in endometrial cancer are limited. At the 2018 ASCO annual meeting, GRAIL biotechnology reported that the sensitivity of ctDNA detection for EC patients was lower than 10%, but its specificity was as high as 95% [17]. In 2010, Dobrzycka [15] also described that ctDNA was detected in the bloodstream of merely 18% of patients. Therefore, as a means of early screening for endometrial cancer, this technique is still marginally insufficient.

### Disease surveillance of EC

Some scholars investigated the ctDNA levels in 9 patients with high-risk EC [18] and demonstrated that ctDNA could be detected in 67% of baseline plasma samples, and the levels of cfDNA and ctDNA in relapsed patients were higher than those in non-relapsed patients. At the same time, they also evaluated the correlation between ctDNA and tumor recurrence and described that the sensitivity and specificity of postoperative ctDNA levels in predicting tumor recurrence were 100% and 83.3%, respectively, with a Kappa index of 0.769; these findings signal that there is a significant agreement between ctDNA detection and tumor recurrence after surgery, and ctDNA is superior to CA125 or HE4 in detecting tumor recurrence. A study conducted in China enrolling 46 patients with EC found [19] that patients with recurrence were negative for ctDNA before the surgery and positive during postoperative follow-up, and clinical recurrence was determined one month after reexamination. The aforementioned results signal that for patients with high-risk EC, ctDNA is a sensitive and specific biomarker for monitoring tumor recurrence, and fluctuations in its level indicate the change of tumor load to reflect the risk of recurrence.

Another study also found [19] that all ctDNA-positive patients before initial treatment became negative after treatment, and ctDNA-negative patients had longer progression-free survival (PFS) and overall survival (OS) [20] times, indicating that ctDNA may be used as an indicator to evaluate treatment efficacy in EC.

Carlos et al. [21] used ddPCR to measure ctDNA levels in 60 EC patients and reported that 56.3% of patients with high-risk tumors were positive for ctDNA at the time of surgery, while only 15.8% of patients with low-risk tumors were ctDNA-positive. In addition, ctDNA levels were higher in EC patients with higher grades and deeper myometrial invasion, signifying that ctDNA can monitor the tumor burden of endometrial cancer patients.

Some researchers also employed ddPCR to detect PIK3CA or KRAS mutations in cfDNA to explore the correlation between ctDNA and EC [22]. The results exposed that ctDNA was significantly correlated with the histopathological type, stage, and grade of EC and was an independent risk factor for lymphatic invasion. Meanwhile, the disease-free survival (DFS) and OS of patients with positive ctDNA before surgery were shorter. Contrastingly, another study [18] reported that ctDNA was not significantly correlated with other clinicopathological features except FIGO stage and lymph node status through multiple pre- and postoperative reexamination of patients with EC; nevertheless, postoperative detection of ctDNA was more predictive of DFS than preoperative detection, indicating that postoperative monitoring of ctDNA is more meaningful than preoperative monitoring in predicting tumor recurrence.

### Risk analysis of recurrence and metastasis

Currently, there is no specific biomarker for the follow-up of EC, and imaging approaches are usually relied on for routine clinical monitoring. Pereira et al. postulated that ctDNA is an independent predictor of the survival rate of patients with ovarian and endometrial cancers. Regular monitoring of ctDNA levels in patients with gynecological tumors can diagnose disease recurrence earlier than CT, and monitoring ctDNA levels after treatment can provide a new predictor of the survival rate of patients [20]. For instance, Tie et al. [23] reported that positive ctDNA in patients with colon cancer following surgery implies that there are small residual lesions in the patient; adjuvant treatment is warranted to eliminate these residual lesions in order to reduce the risk of recurrence and metastasis. A recent small sample study also demonstrated that [24] ctDNA can detect the recurrence or progression of EC approximately 2.5 months (1–8 months) earlier than conventional imaging techniques. Herein, ctDNA could be detected in patients diagnosed with stage I EC who subsequently relapsed, and an elevated level was representative of disease progression, as confirmed by imaging techniques, and can accurately reflect radiotherapy response in patients with recurrent EC undergoing chemotherapy. However, real-world prospective large sample studies are limited. Indeed, studies on the correlation between ctDNA and recurrence and disease progression of EC are limited, and the sample size is not large; this warrants further investigations.

### Treatment options

The results of a large-scale phase II keynote-158 study showed that [25] PD-1 inhibitors have superior efficacy in tumor patients with MSI-H/dMMR, and ctDNA can identify the MSI-H status in recurrent EC [24]. The guidelines also recommend determining the MSI status of EC patients so that ctDNA can be utilized to guide the use of PD-1 inhibitors [26]. At the same time, the lack of specific biomarkers to monitor the disease activity of endometrial cancer indicates that EC-specific ctDNA detection has promising clinical value, especially in monitoring recurrence and guiding future treatment options.

The feasibility of ctDNA detection for recurrent EC was validated through a non-interventional cohort study of 18 patients with EC [27]. The results showed that compared with clinical examination, ctDNA detection was more physiologically and psychologically acceptable, and subjects overwhelmingly preferred blood examination over pelvic examination. CtDNA monitoring to determine EC recurrence seems acceptable to patients, and for many patients, it may be more suitable for clinical evaluation.

An important limitation of ctDNA is the need for a sufficient concentration of ctDNA to produce accurate results. In addition, these detection methods also rely on known mutations of each cancer type, limiting their use for whole cancer detection [3].

### Cell-free non-coding RNA.

NcRNAs include long ncRNAs and short ncRNAs. As is well established, they can play a dual role as a carcinogen or by inhibiting tumor progression in gynecologic cancers. Some scholars also believe that ncRNAs have great potential in personalized drug therapy targets [28], which can minimize the side effects of systematic therapy and allow different targeted therapies for each subgroup of patients.

MALAT1, HOTAIR, H19, OVAL, CCAT1, etc., in lncRNA can all exert carcinogenic effects in EC [29–33]. Among them, HOTAIR gene expression responds to estradiol in endometrial cancer. When its expression level increases, the metastasis rate increases while the overall survival rate decreases [30]. OVAL is abundantly expressed in type I EC, whereas lowly expressed in type II EC [32], and thus can be used as a potential marker for the pathological classification of EC. CCAT1 can influence the growth of endometrial cancer cells and the expression of growth regulatory genes, thus playing an oncogenic role. Further research is necessitated in the future to explore whether it is a potential therapeutic target for EC [33]. In addition, the high expression of PCAT1 [34] is positively correlated with FIGO stage, myometrial invasion, lymph node metastasis, and shortened overall survival of endometrial cancer and, therefore, may be a promising biomarker for the prognosis of patients with endometrial cancer. A recent study has found that the lncRNA SRA is involved in the proliferation, migration, and invasion of EC cells by increasing the expression of ELF4E-BP1 and activity of Wnt/β-catenin signaling, indicating that SRA may be a novel biomarker to predict the recurrence and prognosis of endometrial cancer, and may also become a promising therapeutic target for endometrial cancer [35].

MiRNAs (microRNAs) in sncRNAs can assist in distinguishing high- from low-grade endometrioid tumors. A previous study [36] enrolling 36 patients with EC and 40 healthy people reported that a high expression level of MiR-182 was significantly associated with high-grade endometrial cancer. Additionally, it was found that the AUC of MiR-200a, MiR-20b, MiR-200c, MiR-205, and MiR-182 was 0.958, while the sensitivity and specificity and positive and negative predictive values were 92%, 89%, 89%, and 91%, respectively, demonstrating the high diagnostic accuracy of miRNA detection for endometrial cancer. Furthermore, a meta-analysis including 82 studies highlighted that MiR may be a prognostic factor for the treatment of EC patients, especially when it is related to prognostic factors such as lymph node status, LVSI, and recurrence-free survival [37].

In 2019, scholars established a risk assessment model composed of LINC00475, LINC01352, miR503HG, KCNMB2-AS1, and LINC01143, according to the Cancer Genome Atlas Database (TCGA). The analysis of the survival curve showed that the area under the curve (AUC) was 0.978, showing favorable risk prediction performance, and thus can be used to guide personalized treatments of EC patients and add prognostic value to the current clinical system [38]. Wu [39] and Liu [40] also established a risk assessment model composed of 11 miRNAs, including miR-4758, miR-876, miR-142, miR-190b, hsa-miR-216, hsa-miR-363, hsa-miR-940, hsa-miR-1301, and hsa-miR-3614, according to the database. Functional enrichment analysis revealed that these miRNAs were correlated with the occurrence and development of cancer and could predict the overall survival rate of endometrial cancer.

In terms of treatment, prior studies have described that [41], after demethylation, miR-34b is identified as an upregulated miRNA, miR‑34B enhances the sensitivity of EC cells to paclitaxel, and miR‐34 and its predicted target gene MET are key targets for the treatment of endometrial cancer. Collectively, these results are helpful for the development of demethylation agents, miR-34b mimics, MET inhibitors, and anticancer drugs.

Although liquid biopsy has been widely used for the diagnosis and monitoring of breast cancer, colorectal cancer, or prostate cancer, ctDNA is also becoming a potential modality to detect the recurrence of numerous types of cancer. Regrettably, it is still limited to gynecological tumors. In the process of surgical stratification and follow-up of patients with moderate, high-risk, and advanced endometrial cancers, liquid biopsy technology can be used to evaluate residual lesions, detect recurrence early, diagnose and treat, select personalized treatment, and monitor relapse of diseases related to drug resistance. However, the value of liquid biopsy-related technologies in endometrial cancer, such as diagnostic and screening tools based on tumor substances in uterine aspirates and prognostic monitoring tools based on tumor substances in circulation such as CTC or ctDNA, still warrants further exploration in large-scale clinical trials. Although there are some challenges, such as a lack of unified standard, large heterogeneity, and imperfect detection methods, the potential of liquid biopsy to guide clinical decision-making remains high and may provide opportunities for individualized treatment of gynecological tumors and reflect its unique clinical value.

## Data Availability

Not applicable.
